# Social Incentive Mechanism Based Multi-User Sensing Time Optimization in Co-Operative Spectrum Sensing with Mobile Crowd Sensing

**DOI:** 10.3390/s18010250

**Published:** 2018-01-16

**Authors:** Xiaohui Li, Qi Zhu

**Affiliations:** Key Wireless Laboratory of Jiangsu Province, School of Telecommunication and Information Engineering, Nanjing University of Posts and Telecommunications, Nanjing 210003, China; 2016010102@njupt.edu.cn

**Keywords:** mobile crowd sensing, incentive mechanism, co-operative spectrum sensing, game theory

## Abstract

Co-operative spectrum sensing emerging as a significant method to improve the utilization of the spectrum needs sufficient sensing users to participate. Existing related papers consider only the limited secondary users in current sensing system and assume that they will always perform the co-operative spectrum sensing out of obligation. However, this assumption is impractical in the realistic situation where the secondary users are rational and they will not join in the co-operative sensing process without a certain reward to compensate their sensing energy consumption, especially the ones who have no data transmitting in current time slot. To solve this problem, we take advantage of the mobile crowd sensing to supply adequate co-operative sensing candidates, in which the sensing users are not only the secondary users but also a crowd of widely distributed mobile users equipped with personal spectrum sensors (such as smartphones, vehicle sensors). Furthermore, a social incentive mechanism is also adapted to motivate the participations of mobile sensing users. In this paper, we model the interactions among the motivated sensing users as a co-operative game where they adjust their own sensing time strategies to maximize the co-operative sensing utility, which eventually guarantees the detection performance and prevents the global sensing cost being too high. We prove that the game based optimization problem is NP-hard and exists a unique optimal equilibrium. An improved differential evolution algorithm is proposed to solve the optimization problem. Simulation results prove the better performance in our proposed multi-user sensing time optimization model and the proposed improved differential evolution algorithm, respectively compared with the non-optimization model and the other two typical equilibrium solution algorithms.

## 1. Introduction

Cognitive radio (CR) [1] has attracted significant attention due to its reliability to identify the underutilized licensed spectrum bands and improve the spectral efficiency [2]. A fundamental task for secondary user (SU) in CR is to sense spectrum and detect whether the primary user (PU) is absent. Once discovering spectrum holes, SUs will access the underutilized spectrum immediately and hence improve the spectrum efficiency. In a word, spectrum sensing is the core function in CR communication where SUs usually split their time slots into two parts: one for sensing and the other for data transmission. Specifically, increasing sensing time guarantees a higher detecting accuracy, yet it may also incur to a decreasing achievable throughput due to the reduction of transmitting time.

Numerous efforts have been done in the optimization of sensing time [3,4,5] to improve the achievable throughput while preventing the PU from harmful interference. Furthermore, owing to the overhead cost in spectrum sensing such as time delay and energy consumption, energy efficiency [6,7,8] is essential and practical to be considered in the issue of sensing time optimization. Both references [6,7] jointly optimize the sensing time as well as the transmission power to maximize the energy efficiency. Furthermore in [7], Haijun also considers the interference mitigation and imperfect hybrid spectrum sensing in the energy-efficient optimization issue. Based on energy harvesting techniques, the joint optimization of sensing interval, sensing energy and transmit energy is studied by Zan [8] for maximizing the long-term average weighed sum of the throughput of the SU at the cost of tolerable interference to the PU.

All above existing works consider only single SU or non-co-operative SUs in the issue of spectrum sensing time optimization. However, on account of the uncertainty factors caused by channel randomness, such as fading and shadowing, the detection performance from individual SU may be sharply degraded. Thus, the multi-user sensing time optimization in co-operative spectrum sensing [9] should be emphatically studied due to its effectiveness and practicability to improve the detection performance where spatially distributed SUs cooperate and make a collaborative decision about the status of the PU. To our best knowledge, merely works have been focused on this issue. Although some papers perform the optimization of co-operative sensing time [10,11], they all assume the sensing time is the same as each other and there are always sufficient co-operative SUs who sense the same channel and transmit data in it. However, this assumption may be irrational and impractical in the realistic situation where only few SUs sense the same channel and transmit in the current time slot, which will result in the degradation of co-operative sensing performance. Furthermore, in reality, co-operative SUs are all individually rational, hence, if they have no data transmitting in the current slot or obtain no corresponding rewards, they will not participate in the co-operative sensing process at the cost of energy consumption.

To solve these problems, Mobile Crowd Sensing (MCS) [12] emerging as a new sensing paradigm can be used to provide sufficient sensing users who are not only SUs in current spectrum sensing system but also any widely distributed individual users equipped with available sensors (such as vehicle sensors, smart phones, tablets). The task assignment problem in MCS is studied in [13] and two task assignment algorithms are proposed based on a greedy strategy. Moreover, in order to stimulate the selfish and rational crowd sensing users including the SUs who have no data transmitting currently to participate in the co-operative sensing process, the incentive mechanism [14] is necessary to be adopted in the mobile crowd sensing paradigm. 

Numerous researches have studied the incentive mechanism in MCS to solve kinds of issues. A repeated game based co-operative incentive mechanism is designed in [15] to model the interactions among participants for completing a recurrent crowd sensing task. References [16,17,18,19] focus on the trustworthiness of crowd sensed data by designing proper incentive mechanisms. Specifically, Haiming in [16] proposes a truth discovery algorithm combing with a payment based incentive algorithm to guarantee the high aggregation accuracy. Maryam in [17] introduces a new statistical metric to quantify crowd-sensed data trustworthiness and designs a vote-based scheme for smart city crowd sensing in [18] to ensure the anchor-based trustworthiness by using only votes of the participating smartphone users. Furthermore, a game based trustworthiness-driven user incentive mechanism is also proposed in [19]. Paper [20] aims at privacy-preserving incentive mechanism by selecting the ones who are more likely to provide reliable data and compensating their costs for both sensing and privacy leakage. References [21,22,23,24,25,26] take advantage of the incentive mechanism to provide large-scale and high-quality data collected from a crowd of widespread individuals sensing users. In [21,22,23], auction based dynamic *monetary incentives* [14] are used to encourage participants to conduct tasks where a variable budget is set for each task and changes over the system conditions. In addition to monetary incentives, the advent of communities and social networks also popularizes the *social incentives* [24,25,26], i.e., the nonmonetary incentives, where the community consists of the users who have social relationship with each other and voluntary users who can obtain the social rewards, such as community membership, reputation, new friends and community recognition, by participating in the sensing tasks. Numerous people join in social networks for various aims: being aware of others’ situation, developing relationships with other members, building reputation and acquiring own benefit by sharing the contributions created by the community. Thus, the non-monetary incentives can take advantage of the infrastructure of social networks to economically guarantee an adequate number of candidate participants. And Kyungsik et al. [27] conducted a survey on social incentives, which has shown that the interactions among members within a community or social network have powerful motivations for widely distributed mobile users to take part in the MCS schemes.

In this paper, we adopt the novel MCS in the co-operative spectrum sensing where the wide crowd of mobile sensors can be the candidates to participate in the sensing process. Furthermore, taking account of the sensing cost and the proceeding development of various social networks, we adopt the social incentive mechanism to motivate the sensing participation of ubiquitous mobile users. These motivated users, defined as sensing helpers (SHs), compose a new sensing coalition to help the sensing requester (SR) detect the status of the PU. Taking account of the sensing consumption (such as storage, energy and computation) related with the sensing time, each co-operative sensing user would adjust own sensing time to maximize the co-operative sensing utility considering both the co-operative detection performance and the global sensing consumption. The main contributions in this paper are listed as follows:

- We apply the advanced MCS into the co-operative spectrum sensing where not only the existing secondary users in current sensing system but also a crowd of widely distributed mobile users equipped with personal sensors can be regarded as the sufficient candidate co-operative sensing users. Furthermore, considering the individual rationality of each social man, we also adopt the social incentive mechanism to motivate the sensing participation of mobile users where a social reward, including community recognition, community membership and new friends and evaluated reputation can be obtained by each SH within the co-operative coalition. Compared with existing researches focusing on the co-operative spectrum sensing, the contributions address the issue that current secondary users may be insufficient and the sensing users will not voluntarily join in the co-operative spectrum sensing coalition due to the increasing sensing consumption.
- We propose co-operative game based multi-user sensing time optimization model where each SH acting as a player adjust its own sensing time strategy to maximize the co-operative sensing utility taking account of both the co-operative detection performance and the global sensing cost. Compared with existing researches considering only one secondary user or non-co-operative users in the issue of sensing time optimization, this contribution comes to be more realistic and can be widely applied into the improvement of the co-operative detection performance by jointly optimizing the sensing time strategies of all co-operative sensing users.
- We adopt an improved differential evolution algorithm to solve the game based multi-user sensing time optimization problem, which has been proven a NP-hard problem with a unique equilibrium strategy profile. A dynamically adjusting differential weight is proposed in the algorithm. Compared with the two typical equilibrium solution algorithms (i.e., the best response dynamic and fictitious play algorithms), the contributed algorithm can obtain a better co-operative utility (i.e., a better sub-optimal solution) due to the capability of searching a larger scale of candidate solutions and preventing trapping in a local optimum.

The paper is organized as follows. Section 2 describes the system model of the co-operative spectrum sensing based on the mobile crowd sensing and social incentive mechanism. A detailed illustration of the multi-user sensing time optimization for the SHs within the co-operative sensing coalition is given in Section 3. Section 4 shows the simulation results and the conclusion is presented in Section 5.

## 2. System Model

**Figure 1:**
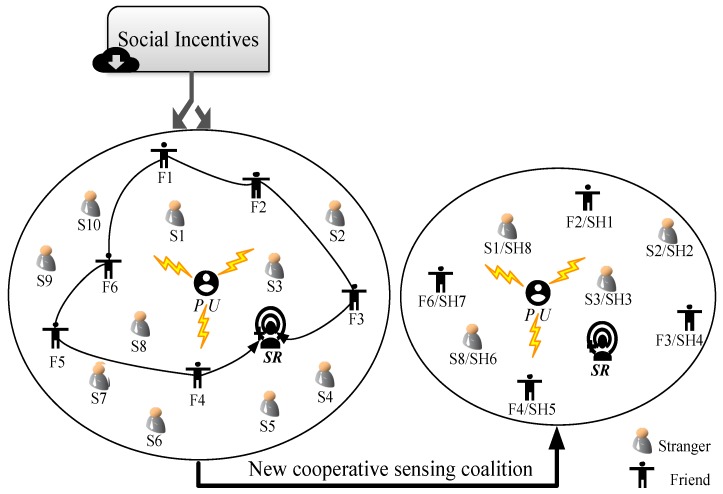
System model.

We design a multi-user co-operative spectrum sensing time optimization model based on the MCS and social incentive mechanism where the members are the widely distributed idle mobile users, including existing SUs and they are motivated by the social incentives, including the friendship, community recognition, community membership and reputation reward. The ones who have been motivated, namely SHs, compose a sensing coalition within that they will adjust their own sensing time to maximize the co-operative sensing utility, which eventually helps the SR guarantee the detection performance and prevents the sensing cost being too high. The interactions among SHs can be modeled as a *co-operative game* [28] in which the players are all SHs and the strategy of each player is the sensing time. The proposed system model is illustrated in Figure 1.

As shown in Figure 1, the PU, SR and a crowd of mobile users, including the friends (i.e., from F1 to F6) within the social circle of the SR and the strangers (i.e., from S1 to S10), randomly distribute in a certain region. Specifically, when a SR needs multiple mobile users helping co-operatively detect the status of PU, it will send out the request both in and out its own social circle. In the social circle of the SR, since they have strong relationship and frequent interactions with the SR, the idle friends among them (i.e., the users F2, F3, F4 and F6) will help the consolidation of friendship and the equivalent returns in future, especially those who have received the assistance of the SR previously. While for the ones out of the social circle, i.e., the strangers form S1 to S10, they will apply for participating in the sensing community considering the acquirement of social rewards including community recognition, community membership and new friends, especially for the ones who has not yet joined in any community or social network. However, some malicious users may also intend to join in the sensing community and consequently attack the sensing data or sensing users. Thus, it is necessary to design a reputation-based selection mechanism, aimed at making a preliminary selection of the strange sensing applicants. Namely, the users who willing to join in the sensing coalition need to firstly send a validation message including its current reputation value to the SR who then perform the selection of candidate sensing users. In the end, the joining helpful friends (i.e., the users F2, F3, F4, F6) and selected strange applicants (i.e., the users S1, S2, S3, S8) compose a new sensing coalition G where the number of SHs is N (specifically, in Figure 1, N=8) and they will conduct a co-operative spectrum sensing to help the SR determine the status of the PU. Table 1 shows the descriptions of crucial notations in the proposed model.

Within the coalition, each SH adjusts its own sensing time strategy to improve the co-operative detection performance and simultaneously prevent the global sensing cost being too high. The energy detection method is adopted. The local detection probability $q_{j}^{d}$ and false alarm probability $q_{j}^{f}$ for SH j within the coalition can be represented by [29]:

(1) $$q_{j}^{d}(t_{j})=Q((\frac{\varepsilon }{\sigma^{2}}-\gamma_{j}-1)\sqrt{\frac{t_{j}f}{2\gamma_{j}+1}})$$

(2) $$\begin{array}{cc} q_{j}^{f}(t_{j}) & =Q((\frac{\varepsilon }{\sigma^{2}}-1)\sqrt{t_{j}f})\\ & =Q(\sqrt{2\gamma_{j}+1}(Q^{-1}({\hat{q}}^{d}))+\sqrt{t_{j}f}\gamma_{j}) \end{array}$$

where the standard Gaussian Q-function is defined as

(3) $$Q(x)=\frac{1}{\sqrt{2\pi }}\int_{x}^{\infty }\exp(-t^{2}/2)dt$$

and ε is detection threshold of energy detector, $\sigma^{2}$ is the variance of circularly symmetric complex Gaussian (CSCG) noise, $t_{j}$ is sensing time of user j, f is the sampling frequency, ${\hat{q}}^{d}$ is a target detection probability and $\gamma^{j}=\frac{{Wh}_{j,PU}}{\sigma^{2}}$ is received signal-to-noise (SNR) over the link from PU to user j, in which the W is the transmission power of PU, $h_{j,PU}$ is the path loss between PU and user j.

We consider, in this coalition, the SR is the head node who receives the local detection results reported by all SHs and make a fusion sensing result about the occupant status of the channel. The co-operative detection probability $Q_{G}^{d}$ and the co-operative false alarm probability $Q_{G}^{f}$ for the coalition, using the OR fusion rule, are given as [29]:

(4) $$Q_{G}^{d}(t)=1-\prod_{j\in G}\left[q_{j}^{d}(t_{j})\cdot q_{j,o}^{e}+(1-q_{j}^{d}(t_{j}))(1-q_{j,o}^{e})\right]$$

(5) $$Q_{G}^{f}(t)=1-\prod_{j\in G}\left[q_{j}^{f}(t_{j})\cdot q_{j,o}^{e}+(1-q_{j}^{f}(t_{j}))(1-q_{j,o}^{e})\right]$$

(6) $$q_{j,o}^{e}=\frac{1}{2}(1-\sqrt{\frac{\gamma_{j,o}}{1+\gamma_{j,o}}})$$

where $q_{j,o}^{e}$ is the error probability due to the fading over the reporting channel between SH j and the head node SR and $t=\left\{t_{1},t_{2},\ldots ,t_{N}\right\}$ is the sensing time profile for all SHs. In the model, we assume that the sensing time $t_{j}$ ranges from zero to T, i.e., $0\leq t_{j}\leq T$, so that the vector space of t can be denoted as $T=\left\{t|t_{j}\in \left[0,T\right],\forall j\in G\right\}$. Note that we assume in this paper the motivated idle mobile sensing users are the ones who have no data transmitting in current slot. They will finish the co-operative sensing process during [0,T] and then report the co-operative detection results by head node to the SR. Furthermore, without loss of generality, the SH who has the highest received SNR is selected as the head node of the sensing coalition, which can effectively decease the error probability when transmitting the co-operative sensing results to the SR. Based on the fusion detection result, the SR will finally decide whether transmit or not in current slot and current channel.

As we can see in Equations (1)–(5), given the number of SHs within the coalition, both the local detection probability and local false alarm probability increase over the sensing time, which hence not only improves the co-operative detection probability but also the co-operative false alarm probability. Furthermore, increasing the sensing time will also incur to growing global sensing energy consumption. Thus, it is imperative for all SHs to co-operatively find an optimal sensing time profile t∈T to make the best trade-off between the co-operative detection performance and the global sensing cost.

In conclusion, the interactions among SHs within the coalition can be modeled as a co-operative game where the player is each SH whose strategy is the sensing time $t_{j},\forall j\in G$. All SHs aim to help the SR acquire a better co-operative detection performance by adjusting their own sensing time, whereas a higher global sensing cost may also follow. Therefore, take account of the energy saving, we design a co-operative sensing utility function taking account of both the profit obtained from the co-operative detection probability

(7) $$V(t)=\lambda \ln(a+bQ_{G}^{d}(t))$$

and the global sensing cost due to the sensing consumption of each SH

(8) $$E(t)=\alpha +{\left(\sum_{j\in G}c_{j}t_{j}\right)}^{\beta }$$

where λ>0, α>0 and β>0 are system parameters, $c_{j}$ is the sensing cost per unit time for SH j. The $\ln(a+bQ_{G}^{d}(t))$ function reflects the diminishing return on the increasing co-operative detection probability. Consequently, combining the (7) and (8), the co-operative sensing utility of the coalition is given as

(9) $$\eta (t)=\frac{V(t)}{E(t)}=\frac{\lambda \ln(a+bQ_{G}^{d}(t))}{\alpha +{\left(\sum_{j\in G}c_{j}t_{j}\right)}^{\beta }}.$$

In this paper, our aim is to incentivize a crowd of idle mobile sensing users to co-operatively help the SR achieve excellent detection performance while also decreasing the global sensing cost as much as possible. In a word, SHs within the sensing coalition will maximize the co-operative utility by seeking for an optimal sensing time profile t. Hence the co-operative game based multi-user sensing time optimization problem can be mathematically formulated as

$$\underset{t\in T}{\max}\;\eta (t)=\frac{\lambda \ln(a+bQ_{G}^{d}(t))}{\alpha +{\left(\sum_{j\in G}c_{j}t_{j}\right)}^{\beta }}$$

(10) $$s.t.Q_{G}^{f}(t)\leq \zeta$$

where ζ is the upper limit of co-operative false alarm probability.

As shown in Equation (10), all SHs within the coalition will jointly sensing to achieve a maximal co-operative utility. The numerator part of η(t) represents the co-operative profits based on the co-operative detection probability. The higher co-operative detection probability, the higher co-operative utility can be achieved. While the denominator part of η(t) shows the impacts of global sensing cost on the co-operative utility, which means that the lower global sensing cost can benefit a higher co-operative utility. Hence in order to obtain a better co-operative utility, all SHs within the sensing coalition will try their bests to decrease the global sensing cost by adjusting each own sensing time strategy. In other word, the maximal optimization of the co-operative utility gives the SHs a force to prevent their global sensing cost being too high and vice versa. Actually, as noted in Section 1, it is just one of the main contributions in our proposed model. Based on above analysis, the co-operative utility can be seen as a portion of the profits for each participating SH, combining with the social reward obtained by join in the co-operative coalition, which will compensate the individual sensing cost. Consequently, the individual utility of each SH can be expressed as the proportional co-operative utility plus obtained social reward and meanwhile subtract its own sensing cost. A proper quantization of social reward will be considered in our further study; thus, a formulaic utility function of each SH can be expressed. 

## 3. Social Incentive Mechanism Based Multi-User Sensing Time Optimization

We model the multi-user sensing time optimization problem as a co-operative game where the idle mobile sensing users (i.e., the ones who have no data transmitting in current time slot) motivated by social incentives and sense together as a coalition in which they optimize their own strategies (i.e., the sensing time) to maximize the co-operative utility consisting of the profit gained from co-operative detection probability and global sensing cost.

### 3.1. Properties and Proofs

**Property** **1.** The proposed co-operative game based multi-user sensing time optimization model is individual rational both for the SR and each SH within the sensing coalition.

**Proof.**  In game theory, individual rationality is one of the most essential properties, which means that all players are rational and will not perform any voluntary action unless a non-negative utility can be obtained. Based on the system model, in our proposed model, for the SR, it can get a better detection performance, namely, a higher detection probability given the upper limit of false alarm probability by taking advantaging of the social incentive mechanism to motivate a crowd of sensing participants. Hence a profitable utility can be obtained. While for the SHs joining in the co-operative sensing coalition, we design an incentive mechanism to provide them with social profits, including the friendship, community recognition, community membership and the elevated reputation value when finishing the sensing process. These social profits will benefit them by getting timely sensing helps once they need conduct the co-operative sensing tasks in the future. Furthermore, the elevated reputation value will assist them to successfully join in other coalition and thus get much more profits as the compensation for their sensing cost. Consequently, there also exists a positive reward for each SH. In conclusion, the proposed co-operative game based model proves to be individual rational.

**Definition** **1.** *Reduction algorithm [30] is an algorithm for transforming one problem into another problem. The* P<=Q *shows that the problem* P *is reducible to problem* Q*, or* Q *is the reduction from* P*, which also means that the problem* Q *is at least as hard as problem* P*.*

**Definition** **2.** The travelling salesman problem (TSP) is illustrated as the following question: Given a list of cities and the distances between each pair of cities, what is the shortest possible route that visits each city exactly once and returns to the origin city.

**Property** **2.** *The proposed multi-user sensing time optimization problem in co-operative spectrum sensing comes to be a combinatorial optimization problem and the finding of globally optimal solution* $t^{opt}$ *is NP-hard.*

**Proof.**  Reduction algorithm can be leveraged as an effective method to prove the proposition 1. We define the problem P as the TSP, which has already proved an NP-hard problem. And the problem Q is regarded as the proposed energy-efficient joint sensing time optimization problem. Firstly, we construct a new mathematical model for the TSP problem where the inputs are N cities and N−m−1 optional distances for each city (i.e., the distances between current city and remaining unvisited cities) m is the number of visited cities ranging from 0 to N−1. And the output is an optimal distance distribution rule for each city, namely, assigning an optimal distance for each city within its optional strategy set means that the optimal travel route comes into being. Now we model our proposed optimization problem where the input is N SHs and L optional sensing time strategies for each SH (i.e., the discretization of the range $0\leq t_{j}\leq T$). Without loss of generality, we assume that L≥N and the output is an optimal sensing time distribution rule for each SH, namely, assigning an optimal sensing time for each SH within its optional strategy set means that the optimal strategy profile $t^{opt}$ comes into being. Based on above analysis, the proposed optimization problem has the same structure with TSP. And we can transform the input of P as a part of the input of Q, while the output of Q can also be transformed as the output of P. Both the two transformations can be implemented in polynomial time. Thus, we come to the conclusion that problem P is reducible to problem Q, which consequently means that the proposed optimization problem is as least as difficult as TSP and its globally optimal solution is NP-hard to find.

**Definition** **3.** *A set profile* $t^{opt}=\left(t_{j}^{*},t_{-j}^{*}\right)$ *is the equilibrium of the co-operative game, if and only if for each SH* j*,* ∀j∈G*,* $\eta \left(t_{j}^{*},t_{-j}^{*}\right)\geq \eta \left(t_{j},t_{-j}^{*}\right)$*, where* $t_{-j}^{*}$ *is the optimal strategy profile for all SHs except SH* j*.*

According to Definition 3, the equilibrium means that any SH within the coalition cannot improve the co-operative utility, defined in (10), by deviating unilaterally from its current strategy.

**Property** **3.** The proposed co-operative game based multi-user sensing time optimization problem has a unique equilibrium, i.e., the globally optimal solution, which maximize the co-operative sensing of the coalition.

**Proof.**  The existence and uniqueness of the equilibrium certifies that for each SH j, ∀j∈G, given the others’ optimal strategy, i.e., the strategy profile $t_{-j}^{*}$, its own optimal strategy $t_{j}^{*}$ exists and is unique, which can be proved by computing the first and second derivatives of $\eta \left(t_{j},t_{-j}^{*}\right)$ with respect to the strategy $t_{j}$, ∀j∈G. Yet its workload is heavy since that the function $\eta \left(t_{j},t_{-j}^{*}\right)$ is a function of functions about $t_{j}$ and consists of both numerator function and denominator function. For simplify the calculation amount, we calculate the first and second derivatives both of the co-operative detection profit V(t) and global sensing cost E(t) rather than $\eta \left(t_{j},t_{-j}^{*}\right)$ and then obtain an equivalent result.

First, we rewrite the V(t), E(t) and $Q_{G}^{d}(t)$ as follows:

(11) $$V(t)=V(t_{j},t_{-j}^{*})=\lambda \ln(a+bQ_{G}^{d}(t_{j},t_{-j}^{*}))$$

(12) $$E(t)=E(t_{j},t_{-j}^{*})=\alpha +{\left(c_{j}t_{j}+\sum_{\begin{array}{l} k\in G\\ k\neq j \end{array}}c_{k}t_{k}\right)}^{\beta }$$

(13) $$Q_{G}^{d}(t)=1-D_{1}\cdot \left[q_{j}^{d}(t_{j})\cdot q_{j,o}^{e}+(1-q_{j}^{d}(t_{j}))(1-q_{j,o}^{e})\right]$$

where

(14) $$D_{1}=\prod_{\begin{array}{l} k\in G\\ k\neq j \end{array}}\left[q_{k}^{d}\cdot q_{k,o}^{e}+(1-q_{k}^{d})(1-q_{j,o}^{e})\right]$$

(1) The first derivative of V(t) with respect to $t_{j}$ is

(15) $$\frac{\partial V(t_{j},t_{-j}^{*})}{\partial t_{j}}=\frac{\lambda b}{a+bQ_{G}^{d}(t_{j},t_{-j}^{*})}\cdot \frac{\partial Q_{G}^{d}(t_{j},t_{-j}^{*})}{\partial t_{j}}$$

where

(16) $$\begin{array}{cc} \frac{\partial Q_{G}^{d}(t_{j},t_{-j}^{*})}{\partial t_{j}} & =D_{1}\cdot \left[\left(2q_{j,o}^{e}-1\right)\cdot \frac{\partial q_{j}^{d}(t_{j})}{\partial t_{j}}\right]\\ & =-D_{1}\cdot \sqrt{\frac{\gamma_{j}}{1+\gamma_{j}}}\cdot \frac{\partial q_{j}^{d}(t_{j})}{\partial t_{j}} \end{array}$$

According to the definition of $q_{j}^{d}(t_{j})$ in (1), we obtain

(17) $$\frac{\partial q_{j}^{d}(t_{j})}{\partial t_{j}}=-D_{2}\cdot e^{-\frac{{\left(D_{2}\right)}^{2}t_{j}}{2}}\cdot \frac{1}{\sqrt{t_{j}}}$$

where

(18) $$D_{2}=\frac{1}{2}(\frac{\varepsilon }{\sigma^{2}}-\gamma_{j}-1)\sqrt{\frac{f}{2\pi (2\gamma_{j}+1)}}$$

Substituting Equations (17) and (16) to Equation (15) has

(19) $$\frac{\partial V(t_{j},t_{-j}^{*})}{\partial t_{j}}=D_{3}\cdot D_{4}(t_{j})\cdot D_{5}(t_{j})$$

where

(20) $$D_{3}=\lambda bD_{1}D_{2}\cdot \sqrt{\frac{\gamma_{j}}{1+\gamma_{j}}}$$

(21) $$D_{4}(t_{j})={\left(e^{-\frac{{\left(D_{2}\right)}^{2}}{2}}\right)}^{t_{j}}\cdot {\left(t_{j}\right)}^{-\frac{1}{2}}$$

(22) $$D_{5}(t_{j})={\left(a+bQ_{G}^{d}(t_{j},t_{-j}^{*})\right)}^{-1}$$

Based on above analysis, we can get that

(23) $$D_{1}>0,\;D_{2}>0,\;D_{3}>0,\;D_{4}(t_{j})>0,\;D_{5}(t_{j})>0$$

for $\forall t_{j}\in \left[0,T\right]$.

Consequently, we get

(24) $$\frac{\partial Q_{G}^{d}(t_{j},t_{-j}^{*})}{\partial t_{j}}>0$$

(25) $$\frac{\partial V(t_{j},t_{-j}^{*})}{\partial t_{j}}>0$$

which means that the increasing sensing time of SH j also result in the increasing profit obtained from the co-operative detection probability.

(2) According to Equation (19), the second derivative of V(t) with respect to $t_{j}$ can be expressed as

(26) $$\begin{array}{cc} \frac{\partial^{2}V(t_{j},t_{-j}^{*})}{\partial {\left(t_{j}\right)}^{2}} & =D_{3}\cdot \frac{\partial (D_{4}(t_{j})\cdot D_{5}(t_{j}))}{\partial (t_{j})}\\ & =D_{3}\cdot (\frac{\partial D_{4}(t_{j})}{\partial (t_{j})}\cdot D_{5}(t_{j})+\frac{\partial D_{5}(t_{j})}{\partial (t_{j})}\cdot D_{4}(t_{j})) \end{array}$$

where

(27) $$\frac{\partial D_{4}(t_{j})}{\partial (t_{j})}=-\frac{1}{2}e^{-\frac{{\left(D_{2}\right)}^{2}t_{j}}{2}}\cdot {\left(t_{j}\right)}^{-\frac{1}{2}}\cdot ({\left(D_{2}\right)}^{2}+{\left(t_{j}\right)}^{-1})$$

(28) $$\frac{\partial D_{5}(t_{j})}{\partial (t_{j})}=-(a+bQ_{G}^{d}{\left(t_{j},t_{-j}^{*}\right)}^{-2})\cdot b\cdot \frac{\partial Q_{G}^{d}(t_{j},t_{-j}^{*})}{\partial t_{j}}$$

Based on Equation (27), we have

(29) $$\frac{\partial D_{4}(t_{j})}{\partial (t_{j})}<0$$

Substituting the in Equation (24) to Equation (28) has

(30) $$\frac{\partial D_{5}(t_{j})}{\partial (t_{j})}<0$$

Synthesizing in Equations (23), (29) and (30), we obtain

(31) $$\frac{\partial^{2}V(t_{j},t_{-j}^{*})}{\partial {\left(t_{j}\right)}^{2}}<0$$

which means that the increment of the profit $V(t_{j},t_{-j}^{*})$ has a decreasing marginal benefit over the increasing sensing time $t_{j}$.

(3) The first derivative of E(t) with respect to $t_{j}$ is

(32) $$\frac{\partial E(t_{j},t_{-j}^{*})}{\partial t_{j}}=\beta \cdot c_{j}\cdot {\left(c_{j}t_{j}+\sum_{\begin{array}{l} k\in G\\ k\neq j \end{array}}c_{k}t_{k}\right)}^{\beta -1}$$

And we get

(33) $$\frac{\partial E(t_{j},t_{-j}^{*})}{\partial t_{j}}>0$$

which means that increasing sensing time of SH j also result in the increasing global cost.

(4) The second derivative of E(t) with respect to $t_{j}$ is

(34) $$\frac{\partial^{2}E(t_{j},t_{-j}^{*})}{\partial {\left(t_{j}\right)}^{2}}=\beta \cdot (\beta -1)\cdot {\left(c_{j}\right)}^{2}\cdot {\left(c_{j}t_{j}+\sum_{\begin{array}{l} k\in G\\ k\neq j \end{array}}c_{k}t_{k}\right)}^{\beta -2}$$

Without loss of generality, we assume β>1. Hence, we can get

(35) $$\frac{\partial^{2}E(t_{j},t_{-j}^{*})}{\partial {\left(t_{j}\right)}^{2}}>0$$

which means that the increment of the global cost $E(t_{j},t_{-j}^{*})$ also has an increasing marginal benefit over the increasing sensing time $t_{j}$.

Based on the analysis in parts (1)–(4), we arrive at the following conclusion: with the increasing sensing time of SH j, the increment of the profit $V(t_{j},t_{-j}^{*})$—i.e., the first derivative of $V(t_{j},t_{-j}^{*})$ with respect to $t_{j}$—has a decreasing marginal benefit; while the increment of the global $E(t_{j},t_{-j}^{*})$—i.e., the first derivative of $E(t_{j},t_{-j}^{*})$ with respect to $t_{j}$—has an increasing marginal benefit. Specifically, given the number of SHs within the coalition, both the profit and global sensing cost will be improved with the increasing sensing time strategy but when the sensing time goes up to a certain value, the increasing speed of the obtained profit becomes slower than the global sensing cost. In the end, there will always exist an optimal sensing time strategy profile $t^{opt}$ where the co-operative sensing utility increases up to its maximum and then will decreases. Eventually we proved that the globally optimal solution, i.e., the unique equilibrium, exists in the proposed co-operative game based multi-user sensing time optimization problem.

### 3.2. Algorithm Descriptions

Based on above analysis, the social incentive mechanism based multi-user sensing time optimization in co-operative spectrum sensing can be modeled as a co-operative game where the unique equilibrium has proven to be existent. In recent literature, several learning algorithms emerges as the typical methods to achieve the equilibrium in game theory, such as best response dynamic [31] and fictitious play [32]. However, all these algorithms have the inclination to be trapped in an undesirable equilibrium. Recently, the *Differential Evolution* (DE) [33] has attracted significant attention in many researches [34,35] due to its excellent property of exploring the globally optimal solution. DE is well suited for multidimensional real-valued optimization problems and can search very large spaces of candidate solutions. Thus, aiming for the best solution possible, we adopt an improved DE algorithm into our proposed model where the initial search points are multiple and widely selected in the feasible region of $t_{j}$ for each SH.

The details about the proposed improved DE algorithm are illustrated in Algorithm 1, which consists of the initialization, mutation operation, crossover operation, greedy selection and final output. In the initialization part (i.e., line 1 to line 6), the population is defined as the search scale of candidate solutions and the dimension means the number of SHs in the sensing coalition. Firstly, an NP∗N initial sensing time strategy matrix is formed in line 4. Then based on the mutation operation (i.e., line 9), a son-generation strategy (i.e., the son) is educed for each individual. And it will be seen as the next-1 strategy if the proper scale of sensing time is satisfied (i.e., line 10 and 11). Otherwise a randomly generated strategy will emerge as the next-1 strategy (i.e., line 13). Moreover, in the part of mutation operation, we innovatively improve the differential weight as a dynamic parameter

(36) F=r∗rand(0,1)

where r is a multiplication factor. This improved differential weight evaluates the capacity of exploring a larger search space and decreases the risk of trapping into local maximum. During the crossover operation part, a crossover probability CR is used to decide whether the next-2 strategy is directly the next-1 generation strategy or the initial sensing time strategy (i.e., line 19 to line 23). Based on above results, a greedy selection operation is utilized to select the optimal sensing time strategy profile (i.e., the optimal row vector in sensing strategy matrix), where the $t_{i}^{gm}=\left\{t_{i,j}^{gm},j\in [1,2,\ldots ,N]\right\}$ and $j_{rand}$ is randomly selected among [1,2,…,N]. Specifically, if the next-2 strategy can obtain a better co-operative utility than the initial sensing strategy for each individual, it will be selected. Otherwise the later will be selected. And then an updated sensing time strategy matrix is formed, in which the sub-optimal row vector will be selected as the optimal sensing time strategy profile during the current generation (i.e., the notion gm), if it can achieve the highest co-operative utility. Finally, in the output part, all the optimal sensing time strategy profiles in Gm generations will be sorted in descending order. Then starting from the first one, these profiles will be checked whether they satisfy the threshold of the co-operative false alarm probability or not. The sensing time strategy profile is not regarded as the optimal one of the whole Gm generations until the threshold condition is reached.

**Algorithm 1.** Improved DE based energy-efficient joint sensing time optimization.**Input:** Population: NP; Dimension: N; Generation: Gm**Initialization:**  1. gm←1; $t_{min}\leftarrow 0$; $t_{max}\leftarrow T$
  2. **for**
i=1
**to**
NP**, do**  3.   **for**
j=1
**to**
N**, do**  4.    $t_{i,j}^{gm}=t_{min}+rand(0,1)\cdot (t_{max}-t_{min})$;  5.   **end**  6. **end**  **While**
gm≤Gm**, do****Mutation Operation:**  7. **for**
i=1
**to**
NP**, do**  8.  **for**
j=1
**to**
N**, do**  9.    $son=t_{x_{1},j}^{gm}+F\cdot (t_{x_{2},j}^{gm}-t_{x_{3},j}^{gm})$**,**
      $\forall x_{1},x_{2},x_{3}\in \left[1,2,\ldots ,NP\right]$, $x_{1}\neq x_{2}\neq x_{3}$;   10.    **if**
0<son<T,   11.      $t_{i,j}^{gm}\_next\_1=son$;  12.    **else**
  13.      $t_{i,j}^{gm}\_next\_1=t_{min}+rand(0,1)\cdot (t_{max}-t_{min})$;  14.    **end**  15.  **end**  16. **end****Crossover Operation:**  17. **for**
i=1 to NP, **do**  18.  **for**
j=1
**to**
N**, do**  19.    **if**
CR≥rand(0,1)
**or**
$j=j_{rand}$**,**  20.      $t_{i,j}^{gm}\_next\_2=t_{i,j}^{gm}\_next\_1$;  21.    **else**
  22.      $t_{i,j}^{gm}\_next\_2=t_{i,j}^{gm}$;  23.    **end**  24.  **end**  25. **end****Greedy Selection:**  26. **for**
i=1 to NP, **do**  27.   **for**
j=1
**to**
N**, do**  28.     **if**
$\eta (t_{i,j}^{gm}\_next\_2)>\eta (t_{i,j}^{gm})$
  29.       $t_{i,j}^{gm}\leftarrow t_{i,j}^{gm}\_next\_2$;  30.     **else**  31.       $t_{i,j}^{gm}\leftarrow t_{i,j}^{gm}$;  32.     **end**  33.   **end**  34.   compute $\eta (t_{i}^{gm})$;  35. **end**  36. ${\left(t^{gm}\right)}^{opt}\leftarrow \underset{t_{i}^{gm}}{argmax}\eta (t)$, i=1,2,…,NP;  37. gm←gm+1;  **end**  **Output:** The best strategy profile $t^{opt}$  38. Sort $\eta ({\left(t^{gm}\right)}^{opt})$, gm=1,2,…,Gm in descending order and extract all of the corresponding Generation indexes into the vector best={l|l∈[1,Gm]}
  39. **for**
z=1
**to**
Gm**, do**
  40.   **if**
$Q_{G}^{f}(t^{best(z)})<\zeta$
  41.     $t^{opt}\leftarrow t^{best(1)}$;  42.     break;  43.   **else**  44.    z←z+1;  45.   **end**  46. **end**  47. Obtain the best sensing time strategy profile $t^{opt}$


## 4. Simulation Results

To evaluate the performance of the proposed algorithm, we set up the network topology as follows. The SR are located in a 5 km × 5 km region where the randomly distributed SHs, namely the helpful friends and selected strange applicants, are motivated and compose a new co-operative sensing coalition to detect the status of a PU and maximize the co-operative sensing utility by adjusting their own sensing time strategies. Firstly, in this section, the performances of our proposed algorithm with different simulation parameters are illustrated respectively. Then we compare our proposed multi-user sensing time optimization model with the non-optimization model in which the sensing users perform the co-operative sensing process with just initial sensing time. Furthermore, the proposed improved differential evaluation algorithm is compared with the best response dynamic and fictitious play algorithms to prove that a better optimization solution can be obtained in our algorithm. Note that all the simulation results are obtained under the constraint of the co-operative false alarm probability as defined in Equation (10). Refer to [7], the transmission power of PU W is set as 0.2 w; upper limit of sensing time T is 100 ms; sampling frequency f is 6 MHz and the noise level $\sigma^{2}$ is −10 dBm. We also assume that the sensing cost $c_{j}$ subjects to uniformly distribution ranging from 0 to 1. And the channel gain $h_{j,PU}$ for each SH is set as exponentially distributed with mean value as 0.1. Other simulation parameters are listed in Table 2. The simulation results are achieved in Matlab R2012 environment and are available for the reader to review the performance results.

### 4.1. Performance with Different Parameters

**Figure 2:**
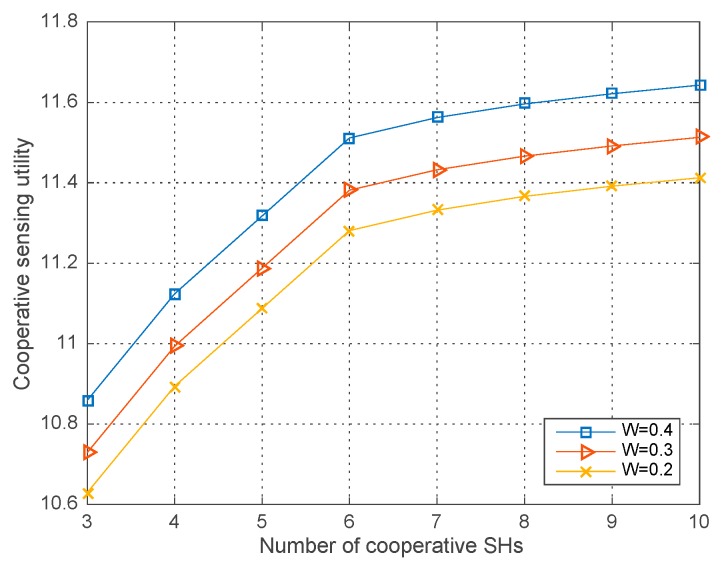
Co-operative sensing utility vs. the number of co-operative SHs with different W.

**Figure 3:**
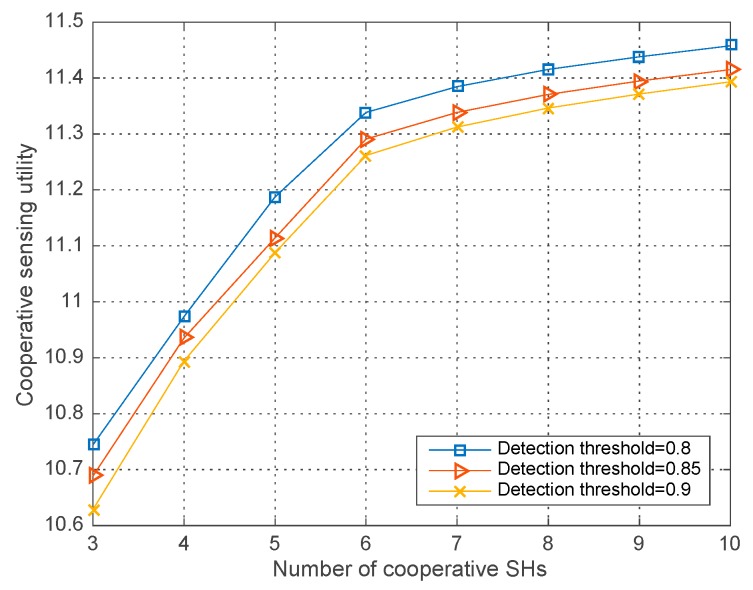
Co-operative sensing utility vs. the number of co-operative SHs with different ε.

**Figure 4:**
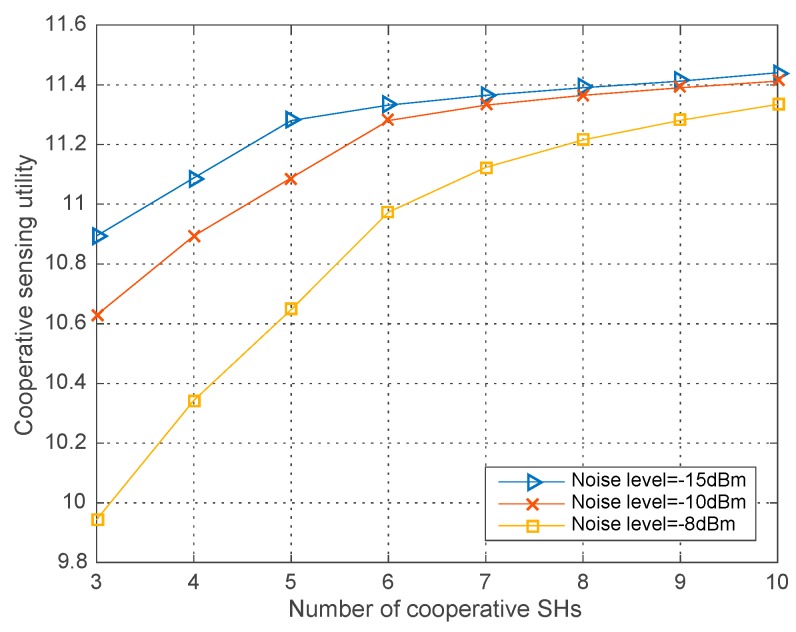
Co-operative sensing utility vs. the number of co-operative SHs with different $\sigma^{2}$.

Figure 2, Figure 3 and Figure 4 respectively illustrate the co-operative sensing utility in our proposed algorithm versus the number of SHs with different simulation parameters, in which the average runs are 200 in each round of simulation. As shown in the Figure 2, Figure 3 and Figure 4, the co-operative sensing utility increases with the number of SHs within the co-operative sensing coalition. Yet the radio of increase is diminishing, which is mainly caused by the faster growing global sensing cost and decelerating growth of the profit with the increasing number of SHs.

Figure 2 shows the co-operative sensing utility with the transmission power of PU W=0.2, 0.3, 0.4 and ε=0.9, $\sigma^{2}=-10$ dBm. We observe that the co-operative sensing utility increases with the PU’s transmission power given the number of co-operative SHs. The reason is that given the channel gain and noise level, the higher PU’s transmission power bring about the higher SH’s received SNR, i.e., $\gamma_{j}=\frac{Wh_{j,PU}}{\sigma^{2}}$. Consequently, the co-operative detection probability will be improved and thus the co-operative sensing utility increases when the global sensing cost remains unchanged.

In Figure 3, the co-operative sensing utility curves with different detection thresholds ε=0.8, 0.85, 0.9 and W=0.2, $\sigma^{2}=-10$ dBm are illustrated. We observe that given the number of co-operative SHs, the co-operative sensing utility decreases with the increasing detection threshold. The reason is that the lower detection threshold of energy detector, the higher detection probability each SH can get, which consequently brings about more profit and hence the co-operative sensing utility increases with the fixed global sensing cost. We can also draw above conclusion in Equation (1) where a higher ε gives rise to a smaller Q-function value and so as to a smaller detection probability obtained for each SH.

Figure 4 shows the co-operative sensing utility with different noise level $\sigma^{2}=-15$, −10, −8 dBm and W=0.2, ε=0.9. As we can see that given the number of co-operative SHs, the higher level of the noise, the lower co-operative sensing utility. The reason is that the high noise level can incur to the degrading received SNR and decreasing detection probability for each SH. In the end, the lower co-operative detection probability results in the lower co-operative sensing utility when the global sensing cost remains fixed.

### 4.2. Model Comparison

**Figure 5:**
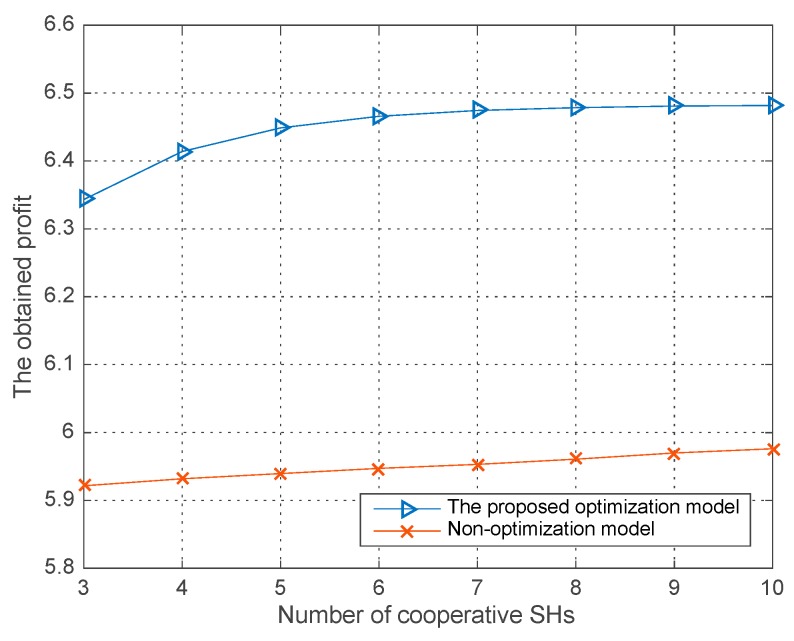
The obtained profit vs. the number of co-operative SHs in different models.

**Figure 6:**
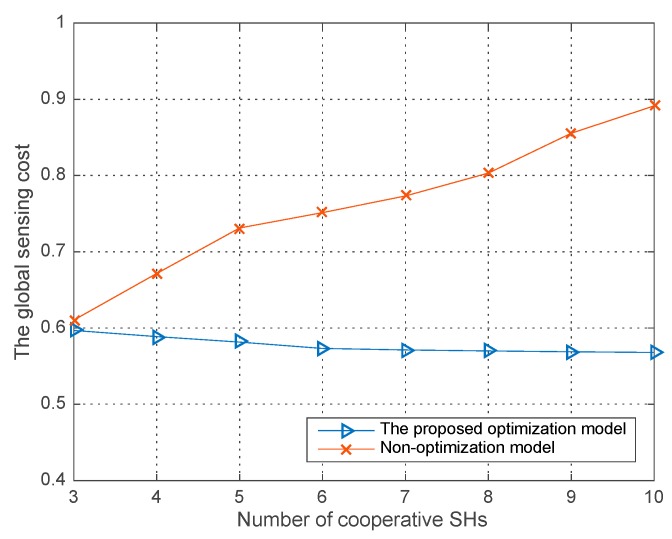
The global sensing cost vs. the number of co-operative SHs in different models.

**Figure 7:**
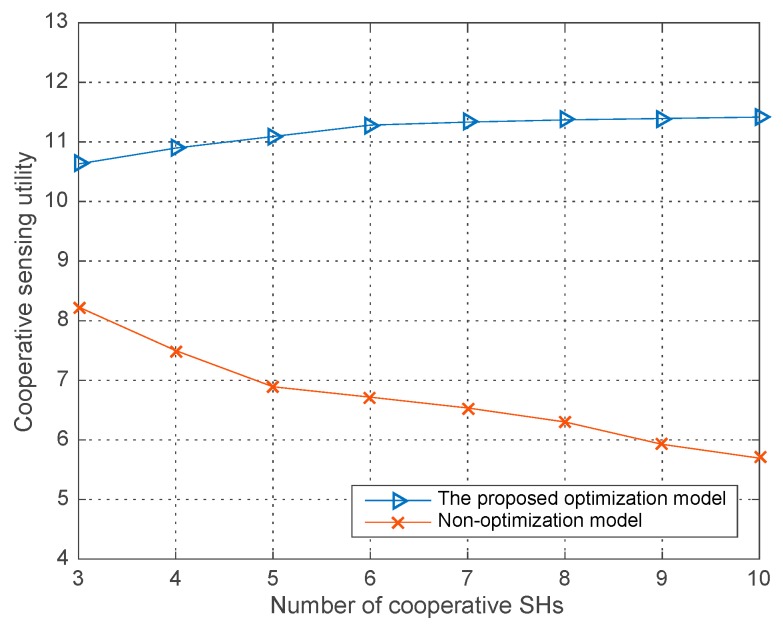
Co-operative sensing utility vs. the number of co-operative SHs in different models.

Figure 5, Figure 6 and Figure 7, illustrates the performances both in our proposed joint sensing time optimization model and the non-optimization model where each SH performing the sensing process just with the initial sensing time allocated in our improved differential evolution algorithm. The average runs are 200 in each round of simulation in the three figures. Specifically, in the non-optimization sensing model, each SH has a fixed sensing time, which results in a fixed co-operative detection performance and global sensing cost in a single trial. While in our proposed sensing time optimization model, given the initial sensing time for each SH, they all optimize their own sensing time in following steps for a better co-operative detection performance and lower global sensing cost—i.e., an optimal co-operative sensing utility. The parameters are listed in Table 1.

Figure 5 and Figure 6 respectively show the obtained profit and global sensing cost versus the number of co-operative SHs in two models. As we can see in Figure 5, the obtained profit increases with the number of co-operative SHs for the reason that a newly participating SH can make a contribution to the co-operative detection probability. Moreover, given a number of co-operative SHs, we can also observe that our proposed sensing time optimization model can obtain a better profit with the dynamic adjusts of each SH’s sensing time strategy in the improved differential evolution algorithm.

Figure 6 shows the global sensing cost versus the number of co-operative SHs in the two models. We observe that without the optimization process for the sensing time of each SH, the global sensing time cost will rapidly increase with the number of co-operative SHs. While for our proposed model, compared with the fixed sensing time of each SH in non-optimization model, the dynamic optimization process can even decrease the global sensing cost with the increasing number of co-operative SHs.

In Figure 7, the co-operative sensing utility curves are illustrated versus the number of co-operative SHs. As shown in the figure, our proposed joint sensing time optimization model can obtain a better co-operative sensing utility compared with the non-optimization model, which can also be known from the Figure 5 and Figure 6. Especially in the Figure 7, if without the optimization of the SHs’ sensing time strategies, the co-operative sensing utility will even decrease with the number of co-operative SHs. In conclusion, all of simulation results in this part demonstrate the advantage of our proposed join sensing time optimization model and the improved differential evolution algorithm.

### 4.3. Algorithm Comparison

**Figure 8:**
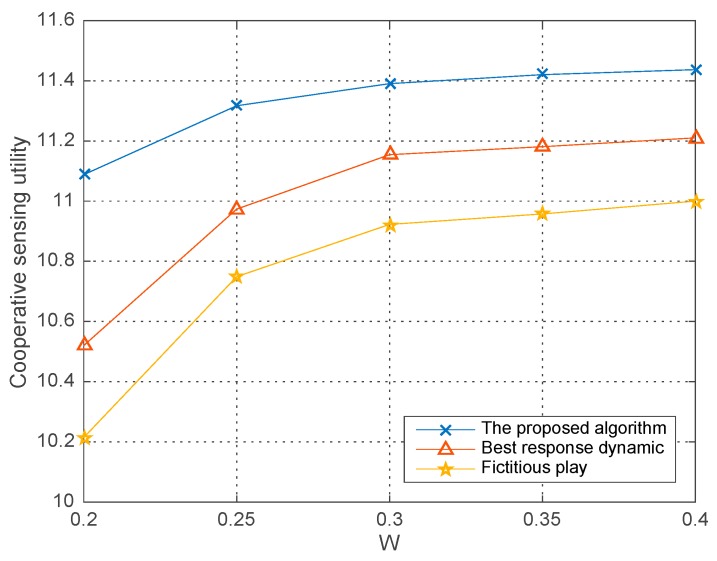
Co-operative sensing utility vs. W in different algorithms.

**Figure 9:**
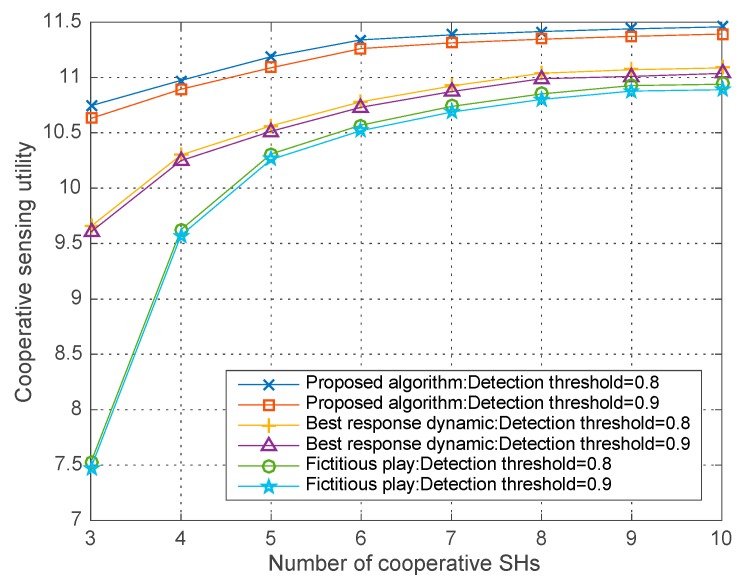
Co-operative sensing utility vs. the number of sensing users with different ε in different algorithms.

**Figure 10:**
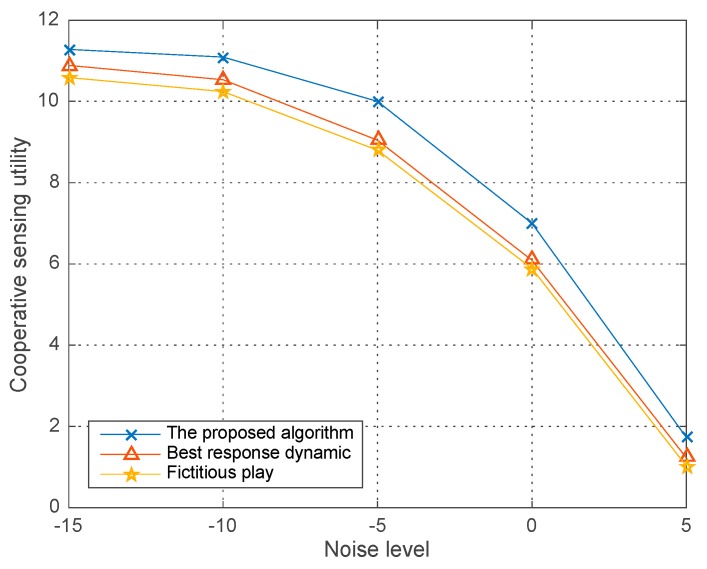
Co-operative sensing utility vs. $\sigma^{2}$ in different algorithms.

**Figure 11:**
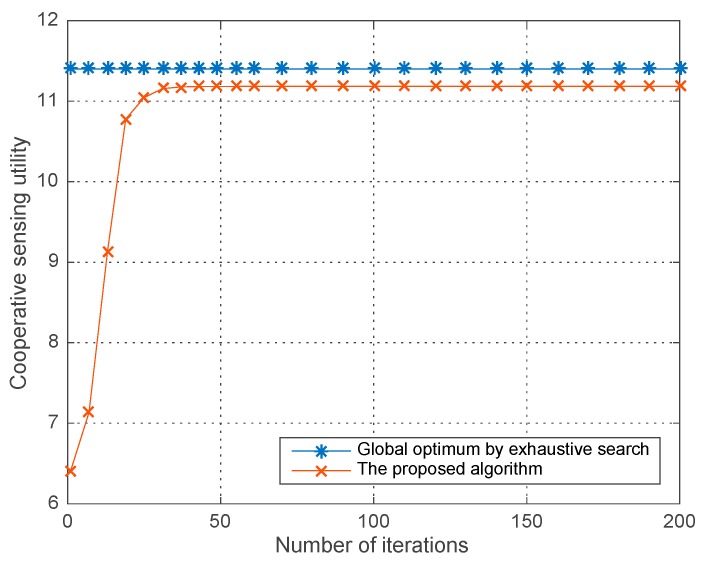
Co-operative sensing utility obtained by proposed algorithm and exhaustive search.

In this part, we illustrate the comparison between our adopted improved differential evolution algorithm and the other two typical equilibrium solution algorithms, i.e., the best response dynamic algorithm and fictitious play algorithm. The co-operative sensing utility as the compared performance with kinds of parameters are demonstrated in Figure 8, Figure 9 and Figure 10 respectively, in which the average runs are 200 in each round of simulation. And Figure 11 further shows the feasibility and confidence of our simulation results by comparing the sub-optimal solution in our proposed algorithm with the global optimum by exhaustive search.

Figure 8 shows the co-operative sensing utility changing with the transmission power of the PU in different algorithms where the number of co-operative SHs are N=5 and ε=0.9, $\sigma^{2}=-10$ dBm. We observe that given the PU’s transmission power, our proposed improved differential evolution algorithm can obtain better co-operative sensing utility than the other two algorithms. The reason is that massive initial search points, i.e., the population and the adjusting differential weight prevents the algorithm plunging into an undesirable local optimum and hence improve the performance.

In Figure 9, we analyze the co-operative sensing utility with different detection threshold ε=0.8, 0.9 and W=0.2, $\sigma^{2}=-10$ dBm in the three algorithms. As shown in the Figure 10, given the number of co-operative SHs, co-operative sensing utility in all three algorithms decrease with the detection threshold. Furthermore, we can see that our proposed algorithm has better performance than the other two algorithms in both situations that ε=0.8, 0.9. This result proves that the proposed improve differential evolution algorithm can converge to a better solution for the modeled NP-hard optimization problem.

Figure 10 illustrates the co-operative sensing utility versus the noise level ranging from −15 dBm to 5 dBm in different algorithms given the PU’s power W=0.2 and detection threshold ε=0.9. We observe that the co-operative sensing utility decreases over the increasing noise level in all the three algorithms. Meanwhile the curves in the figure also demonstrates that a better performance, i.e., a better optimal solution can be obtained in our proposed algorithm compared with the two typical algorithms seeking for the equilibrium in game theory based optimization problem.

Figure 11 compares the sub-optimal utility obtained by our proposed algorithm and the global optimum by exhaustive search, in which the number of SHs within a coalition is N=5 and W=0.2, $\sigma^{2}=-10$ dBm, ε=0.9. As shown in the figure, our proposed algorithm can achieve a near-global optimum solution which eventually proves the feasibility of the proposed improved differential evolution and the confidence of our simulation results. Integrating the results in Figure 8, Figure 9, Figure 10 and Figure 11, we demonstrate that our proposed algorithm can obtain a better sub-optimal solution compared with the other two typical algorithms, i.e., the best response dynamic and the fictitious paly algorithm. And moreover, the obtained sub-optimal is quite approximate to the global optimum solution.

## 5. Conclusions

In this paper, we design a mobile crowd sensing based co-operative spectrum sensing model where the sensing users are a crowd of widely distributed mobile sensing users equipped with kinds of sensing devices (smartphones, vehicle sensors, tablets) and they are motivated by the social incentives, including the friendship, coalition membership, coalition recognition and the evaluated reputation, which can emerge as a kind of reward to compensate their sensing cost. The motivated mobile sensing users apply for joining in the sensing coalition by reporting their current reputation to the SR so that the malicious users can be prevented. These selected sensing users, named as sensing helpers (SHs) form a new sensing coalition and will co-operatively help the SR to detect the status of the PU. The interactions among the SHs are modeled as a co-operative game where the strategy of each player is sensing time and they will co-operatively adjust it to maximize the co-operative sensing utility. Thus, an energy efficient join sensing time optimization is proposed, which considers both the profit obtained from co-operative detection probability and the global sensing cost. Furthermore, the constrain of the co-operative false alarm probability is also taken into account. We prove that the optimization problem is a NP-hard problem with a unique equilibrium solution. Hence in order to find a better sub-solution, we adopt an improved differential evolution algorithm where a large space of candidate solutions can be searched and the differential weight is dynamic. Simulation results illustrate the performance of proposed algorithm changing with different parameters. The comparison between our proposed joint sensing time optimization model and non-optimization model has shown the better profit and lower global sensing cost, i.e., higher co-operative sensing utility, in our proposed model. Furthermore, compared with the typical best response dynamic and fictitious play algorithms in game theory, our proposed improved differential evolution also demonstrates a better co-operative sensing utility.

For the future work, we will take account of the privacy protection in current model to protect the crucial individual information during the selection process of the sensing users. It’s also important to design a mechanism to prevent the candidate malicious users reporting fake statement about their reputation, namely, the truthfulness guarantee in the game based cooperation.

## Figures and Tables

**Table 1:** Descriptions of crucial notations.

Notation	Description	Notation	Description
G	Sensing coalition	$Q_{G}^{f}$	Co-operative detection probability
N	The number of SHs	$t_{j}$	Sensing time strategy of SH j
$q_{j}^{d}$	Local detection probability of SH j	T	Maximal sensing time
${\hat{q}}^{d}$	Target detection probability	t	Sensing time strategy profile
$q_{j}^{f}$	Local false alarm probability of SH j	T	Vector space of t
$q_{j,o}^{e}$	Error probability form SH j to head node	$c_{j}$	Sensing cost of SH j
$Q_{G}^{d}$	Co-operative detection probability	ξ	Threshold of false alarm probability

**Table 2:** Simulation parameters for the proposed algorithm.

Notation	Value	Description
ε	0.9	Detection threshold
λ	1.5	Parameter of obtained profit
a	10	Parameter of obtained profit
b	10	Parameter of obtained profit
α	0.5	Parameter of Global sensing cost
β	0.5	Parameter of Global sensing cost
NP	100	Population size
CR	0.9	Crossover probability
